# The activation mechanism and antibody binding mode for orphan GPR20

**DOI:** 10.1038/s41421-023-00520-8

**Published:** 2023-02-28

**Authors:** Xi Lin, Shan Jiang, Yiran Wu, Xiaohu Wei, Gye-Won Han, Lijie Wu, Junlin Liu, Bo Chen, Zhibin Zhang, Suwen Zhao, Vadim Cherezov, Fei Xu

**Affiliations:** 1 iHuman Institute, ShanghaiTech University, Pudong, Shanghai, China; 2 School of Life Science and Technology, ShanghaiTech University, Shanghai, China; 3 Departments of Chemistry and Biological Sciences, Bridge Institute, University of Southern California, Los Angeles, CA USA

**Keywords:** Cryoelectron microscopy, Cell signalling

## Abstract

GPR20 is a class-A orphan G protein-coupled receptor (GPCR) and a potential therapeutic target for gastrointestinal stromal tumors (GIST) owing to its differentially high expression. An antibody-drug conjugate (ADC) containing a GPR20-binding antibody (Ab046) was recently developed in clinical trials for GIST treatment. GPR20 constitutively activates Gi proteins in the absence of any known ligand, but it remains obscure how this high basal activity is achieved. Here we report three cryo-EM structures of human GPR20 complexes including Gi-coupled GPR20 in the absence or presence of the Fab fragment of Ab046 and Gi-free GPR20. Remarkably, the structures demonstrate a uniquely folded N-terminal helix capping onto the transmembrane domain and our mutagenesis study suggests a key role of this cap region in stimulating the basal activity of GPR20. We also uncover the molecular interactions between GPR20 and Ab046, which may enable the design of tool antibodies with enhanced affinity or new functionality for GPR20. Furthermore, we report the orthosteric pocket occupied by an unassigned density which might be essential for exploring opportunities for deorphanization.

## Introduction

Orphan G protein-coupled receptors (oGPCRs) are pathologically related to many human diseases such as schizophrenia, type 2 diabetes, hyperactivity, cognitive impairment, brain malformation, Alzheimer’s disease and others^1–7^. Many oGPCRs including the adhesion family (aGPCRs) are constitutively active receptors, presenting additional technical hurdles for deorphanization^8^. Since the report of the first ligand-free orphan GPR52 structure by Lin et al.^2^, several structures of oGPCRs in complex with G proteins in the absence of exogenous ligand stimulation have been determined—such as GPR17-Gi^9^, GPR88-Gi^10^, GPR119-Gs^11^ and aGPCR-G protein complexes^12–15^. All these oGPCRs were activated by either their own motif or an endogenous lipid from the cell membrane: the receptor’s extracellular loop 2 (ECL2) folds into the orthosteric pocket and functions as a built-in agonist for GPR52^2^ and GPR17;^9^ the “stalk” region functions as a tethered agonist for aGPCRs;^12–15^ an unassigned density in the orthosteric pocket may represent a putative endogenous ligand for GPR88;^10^ a lipid molecule occupies the orthosteric pocket responsible for the high basal activity of GPR119^11^.

GPR20 is an orphan receptor with its endogenous ligand remaining unknown^16^. It is expressed in many tissues with notably high expression in the intestine. Previous studies identified GPR20 as a novel non-tyrosine kinase target in gastrointestinal stromal tumors (GIST) given its differentially high expression^17,18^. To date, the only approved treatments for GIST are tyrosine kinase inhibitors (TKI), but patients ultimately experience disease progression most often due to the development of heterogeneous secondary resistance mutations in tyrosine-protein kinase KIT^17^. Recently, Daiichi Sankyo developed a DXd-ADC drug (DS-6157a) derived from an anti-GPR20 antibody (Ab046) to inhibit tumor growth in GIST^17^. However, the drug failed in Phase I clinical trial due to a lack of response. Nevertheless, the potential of developing new antibody or derivatives with higher potency and/or new function on GPR20 would deserve further investigation for the treatment of GIST and other related diseases. A previous study suggested that the intrinsic expression of GPR20 is involved in the regulation of cell proliferation by controlling cellular cAMP levels^19^. Indeed, GPR20 shows high level of constitutive activity in the absence of ligand, leading to continuous activation of its coupled Gi proteins^20^. It is unclear how the high basal activity of GPR20 is achieved, and whether a stimulator is required for G-protein coupling and signal transduction.

Here we report the atomic-resolution structures of the ligand-free human orphan GPR20 in the Gi-coupled states in the absence or presence of the Fab fragment of Ab046 (hereafter named as GPR20-Gi and GPR20-Gi-Fab046 respectively) as well as in the Gi-free state (GPR20-Fab046) using single particle cryo-electron microscopy technique (cryo-EM). These structures reveal a uniquely folded N-terminal α-helix (cap) of GPR20 that might be essential in conferring the receptor’s constitutive activity. Our results provide an integrated understanding of the structure and function of GPR20, which paves the way for uncovering the landscape of structural basis for orphan GPCR’s constitutive activity. Structural analysis of the antibody-binding interface as well as the observation of an unassigned density may present opportunity for developing structure-based tool ligands and antibodies for GPR20 deorphanization.

## Results

### Structure of the ligand-free GPR20-Gi complex

**Fig. 1:**
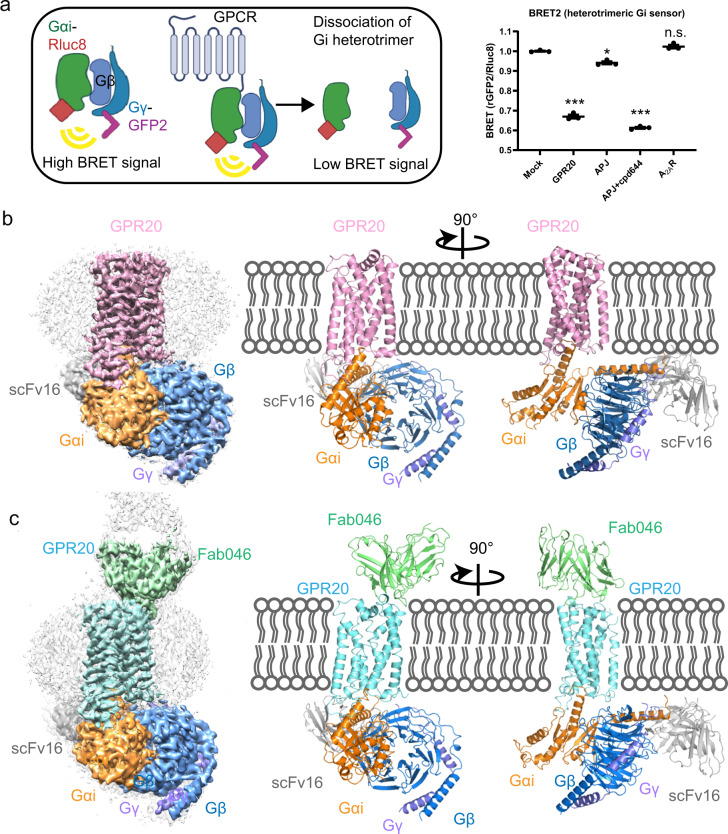
Cryo-EM structure of the ligand-free GPR20-Gi complex. **a** Left, schematic diagram of the bioluminescence resonance energy transfer (BRET2) assay to measure Gi heterotrimer dissociation upon activation. Right, normalized BRET values of HEK293T cells transiently co-transfected with the Gi BRET sensor along with either empty vector (mock control), GPR20, the adenosine A_2A_ receptor (A_2A_R, negative control) or the apelin receptor (APJ, positive control) in the absence and presence of 10 μM agonist cpd644. Data are normalized to the mock control and represented as the mean ± s.e.m. for *n* = 3 biologically independent experiments. Significance was determined by two-way analysis of variance (ANOVA) without repeated measures, followed by Dunnett’s post hoc test (***P < 0.001, *P < 0.05; n.s., not significant). **b** Left, cryo-EM density map of the GPR20-GαiGβGγ-scFv16 complex. Right, two orthogonal views of the cartoon representation of the atomic model of the complex. Color coding is annotated for each protein component. **c** Left, cryo-EM density map of the GPR20-GαiGβGγ-scFv16-Fab046 complex. Right, two orthogonal views of the cartoon representation of the atomic model of the complex. Color coding is annotated for each protein component.

To test whether GPR20 can signal through Gi and understand its high level of constitutive activity^20^, we first performed bioluminescence resonance energy transfer (BRET) assays^21^ to measure G-protein heterotrimer dissociation. The results confirmed the Gi activity by ligand-free GPR20 as shown by the reduced BRET signal compared to the negative controls including the empty vector (mock control) and the adenosine A_2A_ receptor (A_2A_R, which was known to not couple to Gi^22^). It was observed that ligand-free GPR20 showed comparable BRET signal to the apelin receptor (APJ, which was known to couple to Gi^23^) in the presence of an agonist (Fig. 1a). In order to obtain stable GPR20-Gi complex sample amenable for structural investigation by cryo-EM, we tried different strategies (see “Materials and methods” and Supplementary Fig. S1). The final complex was composed of the N-terminal BRIL-fused^24^ wild-type (WT) GPR20, dominant-negative mutant of Gαi1 (containing three mutations: S47N, G203A and A326S^25^), WT Gβ1Gγ2, and a single-chain stabilizing antibody fragment scFv16^26^, which all could be clearly identified by two-dimensional (2D) classification (Supplementary Fig. S1c). Finally, the structure of the ligand-free GPR20-Gi complex was determined at a global resolution of 3.14 Å (Fig. 1b; Supplementary Fig. S1 and Table S1).

To date, there are no ligands available for GPR20; the only reported tool molecule that binds to GPR20 is an anti-GPR20 antibody (Ab046) derived from an ADC drug^17^ for potential GIST treatment. To understand how Ab046 binds and whether it affects the structure and function of GPR20, we also solved the structure of Gi-coupled GPR20 complexed with Fab046. This structure was determined to a global resolution of 3.22 Å with the density maps sufficiently clear to place part of Fab046 (VL and VH of Fab046) into the GPR20-Gi-Fab046 complex (Fig. 1c; Supplementary Fig. S2 and Table S1). The overall structures of the GPR20-Gi-Fab046 complex and GPR20-Gi complex are nearly identical with root mean square deviation (RMSD) values of 0.71 Å for the whole GPR20-Gi complex and 0.70 Å for the receptor alone, which is consistent with our BRET results that Ab046 is a non-functional antibody for GPR20 (Supplementary Fig. S2a).

The overall structures of the GPR20-Gi-Fab046 complex and GPR20-Gi complex show canonical GPCR-G protein complex features; no extra density was observed in the transmembrane helical bundle (7TM) of GPR20 confirming they are both in ligand-free states. We used the structure with higher resolution in transmembrane region (GPR20-Gi-Fab046) for structural illustration in the following analysis unless otherwise noted.

### The N-terminal cap

**Fig. 2:**
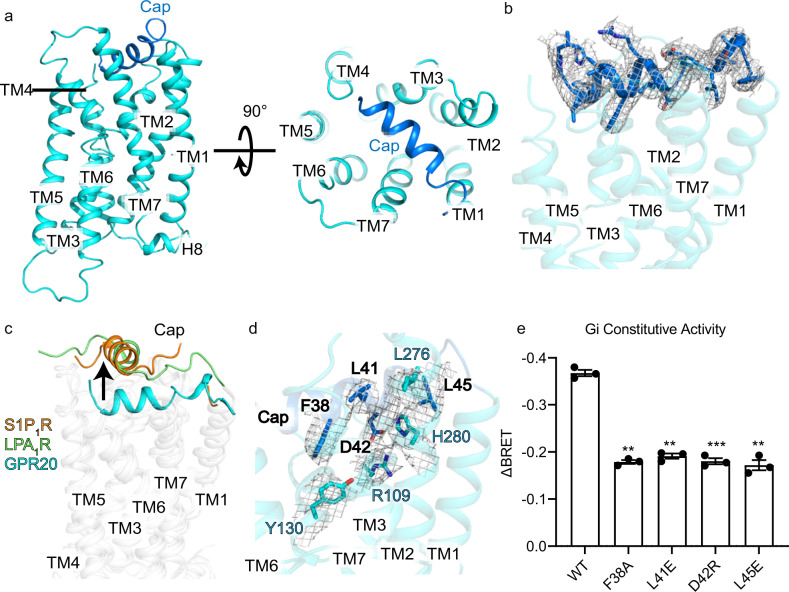
Unique N-terminal region of GPR20. **a** Two orthogonal views of the cartoon representation of GPR20 (cyan) to show the α-helical N-terminal cap (blue). **b** The electron density (gray mesh) of the N-terminal cap region is shown by overlying on the model. **c** Superposition of GPR20 (cyan), S1P_1_R (PDB 7TD3, orange) and LPA_1_R (PDB 7TD0, green) structures to show the respective position of the N-terminal cap in each structure (all aligned to β_2_AR structure (PDB: 3SN6), the Cα atoms of the residues embedded deepest in the pocket were used: F38 for GPR20, Y34 for LPA_1_R and Y29 for S1P_1_R). **d** Magnified view of the N-terminal cap of GPR20. The N-terminal cap and 7TM domain are in transparent blue and cyan cartoon representations, respectively. The side chains of key interacting residues are shown as sticks and overlaid with electron densities (gray mesh). **e** Mutations that may interfere with the conformation of the N-terminal cap impaired GPR20’s basal activity. ΔBRET: the change of bioluminescence resonance energy transfer value (Materials and methods). The ΔBRET was compared between the wild-type (WT) GPR20 and various mutants. Significance was determined by two-way analysis of variance (ANOVA) without repeated measures, followed by Dunnett’s post hoc test (***P < 0.001, **P < 0.01). Data are mean ± s.e.m. (*n* = 3 independent biological experiments).

Overall, the orphan GPR20 adopts a canonical 7TM architecture resembling other class-A GPCRs (Fig. 2a). Remarkably, the N terminal residues 33–49 fold over the top of the receptor forming a unique α-helical cap (Fig. 2a, b). Compared to Sphingosine 1-phosphate receptor subtype 1 (S1P_1_R, PDB: 7TD3)^27^ and Lysophosphatidic acid receptor 1 (LPA_1_R, PDB: 7TD0)^27^, both of which contain a N-terminal helix essential for lipid ligand entry, the N-terminal cap of GPR20 locates deeper in the 7TM core by 9.5 Å than LPA_1_R and S1P_1_R (the Cα atoms of the residues embedded deepest in the pocket were used: F38 for GPR20, Y34 for LPA_1_R and Y29 for S1P_1_R) (Fig. 2c). Structural superposition showed that the positions of ligands in S1P_1_R (sphingosine 1-phosphate, S1P) and LPA_1_R (lysophosphatidic acid, LPA) would clash with the N-terminal cap of GPR20 (Supplementary Fig. S3). The structural characterization hints that the N-terminal cap of GPR20 may behave as a ligand or play important roles in ligand entry and function.

Different to S1P_1_R and LPA_1_R, whose N-terminal helix does not interact with the 7TM region directly, the intramolecular interactions around the N-terminal cap of GPR20 mainly engage TM2, TM3 and TM7 residues: F38^N-term^ is in close contact with Y130^3.32^ in TM3 (superscripts denote Ballesteros–Weinstein numbering^28^), L41^N-term^ forms a hydrophobic interaction with L276^7.32^ in TM7, D42^N-term^ forms a salt bridge with R109^2.60^ in TM2, and L45^N-term^ is in close contact with H280^7.36^ in TM7 (Fig. 2d). Mutagenesis and cellular functional assays showed that mutating the single key residues (F38^N-term^A, L41^N-term^E, D42^N-term^R and L45^N-term^E) on the N-terminal cap all markedly reduced the basal signaling activity of GPR20 (Fig. 2e). This result confirmed our hypothesis that the uniquely folded N-terminal cap may have a key role in stimulating the basal activity of GPR20.

### The activation mechanism of GPR20

**Fig. 3:**
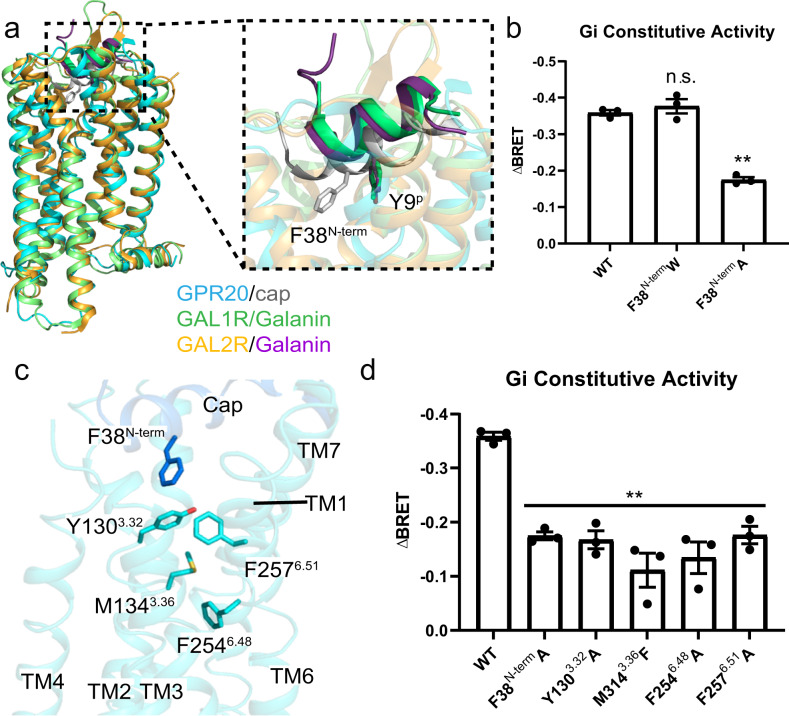
The activation mechanism of GPR20. **a** Left, structural superposition of the ligand-free GPR20-Gi, galanin-bound GAL1R-Gi (PDB 7WQ3) and galanin-bound GAL2R-Gq (PDB 7WQ4) structures. Right, magnified view of the N-terminal cap of GPR20 (gray), galanin in GAL1R (green) and galanin in GAL2R (purple). Key residues are shown as sticks. **b** Constitutive activities of WT GPR20 and F38 mutations are measured by BRET assay. ΔBRET: the change of bioluminescence resonance energy transfer value (Materials and methods). Significance was determined by two-way analysis of variance (ANOVA) without repeated measures, followed by Dunnett’s post hoc test (**P < 0.01, n.s., not significant). Data are mean ± s.e.m. (*n* = 3 independent biological experiments). **c** Four key residues involved in signal transmission from the N-terminal cap to the transmembrane region in GPR20. **d** The effects of key residue (shown in (**c**)) mutations on GPR20’s constitutive activity are measured by BRET assay. Significance was determined by two-way analysis of variance (ANOVA) without repeated measures, followed by Dunnett’s post hoc test (**P < 0.01). Data are mean ± s.e.m. (*n* = 3 independent biological experiments).

A structural comparison of GPR20-Gi-Fab046 complex with all reported GPCR-G protein structures enabled us to examine the conformational features of GPR20 in G protein-coupled state. We found the structure of the ligand-free GPR20-Gi highly resembled that of galanin receptors^29^: galanin-bound GAL1R-Gi (PDB: 7WQ3) with RMSD value for aligned Cα atoms (RMSD_Cα_) of 1.24 Å, and galanin-bound GAL2R-Gq (PDB: 7WQ4) with RMSD_Cα_ of 1.66 Å, although the sequence identities of GPR20 with GAL1R and GAL2R are only 25.1% and 32.2%, respectively (Supplementary Fig. S4). Interestingly, structural superposition of GPR20 and GAL1R/GAL2R reveals that the position of the N-terminal cap of GPR20 overlaps with the position of galanin in GAL1R/GAL2R (Fig. 3a). Additionally, we found that the key residue Y^9P^ on the galanin peptide, which mutation to alanine disrupts galanin’s binding^29^, is very close to F38^N-term^ of GPR20 (Fig. 3a). It was reported that Y^9P^ in galanin engaged extensive hydrophobic network in the receptor to transmit the signal toward the toggle switch^29^. Such an analogy prompts that F38 may be essential for activating GPR20. Indeed, the alanine mutation of F38 significantly decreased basal activity while the tryptophan mutation of F38 had no effect (Fig. 3b).

To understand GPR20’s activation pathway, we focused on the key amino acid F38 in the N-terminal cap and found several hydrophobic residues on TM3 and TM6, including Y130^3.32^, M134^3.36^ and F257^6.51^ that are located below F38 and may transmit the signal to the toggle switch F254^6.48^ through a hydrophobic network (Fig. 3c). Mutagenesis and cellular functional assays showed that the single mutations of these residues impaired basal activity of GPR20 (Fig. 3d). Moreover, we investigated whether the common activation mechanism of class-A receptors can be observed in GPR20. Structural comparison of GPR20-Gi-Fab046 with active and inactive β2 adrenergic receptor (β_2_AR) (PDB: 3SN6^30^ and 2RH1^31^, respectively) reveals an outward movement of TM6 in the G protein-coupled GPR20 relative to inactive β_2_AR (Supplementary Fig. S5a). Several highly conserved micro-switches including the toggle switch (W/F^6.48^), PIF motif (P^5.50^, I^3.40^ and F^6.44^), DRY motif (D^3.49^ and R^3.50^), and NPxxY motif (Y^7.53^) (Supplementary Fig. S5b) in GPR20 resemble the conformation of the active β_2_AR structure. Thus, we conclude that a “signal” initiated by the N-terminal cap might be transmitted through a hydrophobic network toward the toggle switch and activation motifs, thus leading to the active conformation of GPR20 in the ligand-free and G protein-coupled state.

### Structure of the Gi-free GPR20-Fab046 complex

**Fig. 4:**
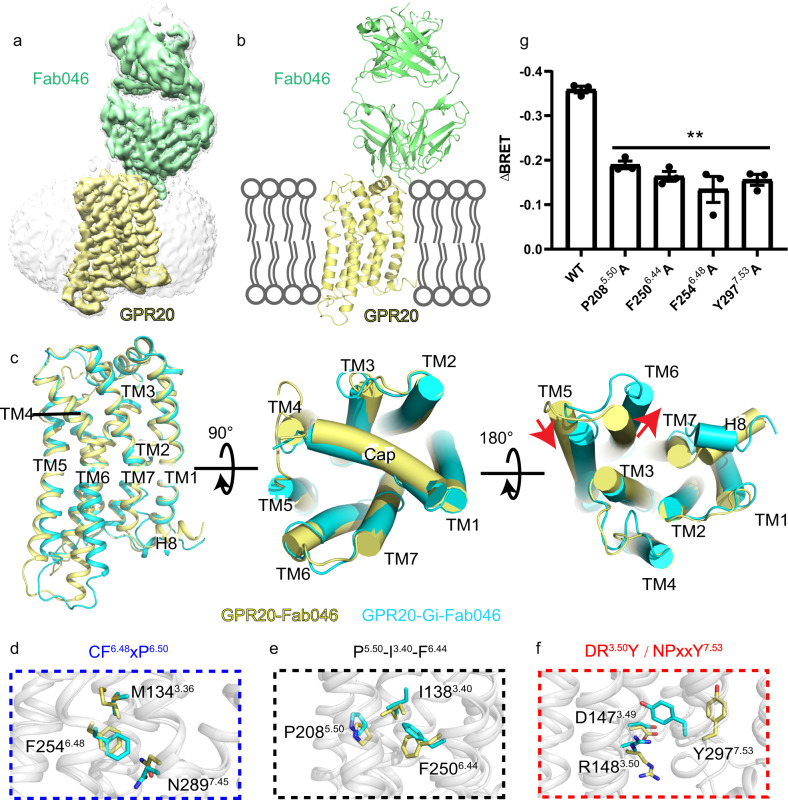
Antibody-bound GPR20 structure. **a**, **b** Cryo-EM density map and atomic models of the GPR20-Fab046 structure. **c** Overlay between GPR20-Fab046 and GPR20-Gi-Fab046 structures in three representative views. Transmembrane helices TM1-TM7 and helix 8 (H8) are labeled. The movements of TM5 and TM6 on the intracellular side are highlighted as red arrows. **d–f** The conformational rearrangement of residues in conserved “micro-switches” upon Gi coupling: CF(W)xP, toggle switch, PIF, DRY and NPxxY motifs. **g** The effects of mutation in micro-switches are measured by BRET assay. Significance was determined by two-way analysis of variance (ANOVA) without repeated measures, followed by Dunnett’s post hoc test (**P < 0.01). Data are mean ± s.e.m. (*n* = 3 independent biological experiments).

To understand the conformational changes induced by G protein coupling, we aimed to determine the structure of Fab046-bound GPR20 without Gi heterotrimer. Therefore, we assembled the purified GPR20 protein with Fab046 (see Materials and methods) for structural investigation. To improve the stability and expression level of WT GPR20 in the absence of co-expressed Gi proteins, two point mutations were introduced on the basis of homology to other class-A receptors: D293^7.49^N^32^ and L139^3.41^W^33^, which were essential for GPR20’s purification. Size-exclusion chromatography (SEC) and SDS-PAGE analysis revealed that GPR20 protein containing the two stabilizing mutations can form a stable complex with Fab046 amenable for cryo-EM studies (Supplementary Fig. S6b). We then determined the GPR20-Fab046 complex structure at a nominal global resolution of 3.08 Å (Fig. 4a, b; Supplementary Fig. S6 and Table S1). This structure is of sufficiently high resolution to allow assignment of the majority of Fab046 residues consisting of the intact light chain and VH + CH1 region of the heavy chain.

Structural comparison of GPR20-Fab046 and GPR20-Gi-Fab046 reveals an outward movement of TM6 for about 7.6 Å (based on Cα of G232^6.26^) and an inward movement of TM5 for about 4.5 Å (based on Cα of L223^5.65^) at the intracellular side in the Gi-coupled relative to Gi-free GPR20 structures (Fig. 4c; Supplementary Fig. S7). These movements are accompanied by structural transformations including the toggle switch (F254^6.48^), PIF motif (P208^5.50^, I138^3.40^ and F250^6.44^), DRY motif (D147^3.49^ and R148^3.50^), and NPxxY motif (Y297^7.53^) (Fig. 4d–f). In agreement with the structural findings, mutagenesis and cellular functional assays showed that mutating a single residue of each of these micro-switches reduced the signaling activity of GPR20 (Fig. 4g). Moreover, through structural comparison of GPR20-Fab046 with active and inactive β_2_AR structures, we found that the GPR20-Fab046 complex was captured in an inactive-like state resembling the conformation of the inactive β_2_AR structure (Supplementary Figs. S8a–c). Closer examination of inactive β_2_AR and GPR20-Fab046 structures revealed different conformations of TMs 5, 6 and 7 that may be partially attributed to the low sequence identity (21.45%) between the two receptors. Also, the position of the ligand carazolol in the inactive β_2_AR forms direct interaction with toggle switch (W286^6.48^) and induces the TM6’s inward movement (Supplementary Fig. S8d). Another possible reason for this difference on TMs 5–7 is that the presence of the N-terminal cap region may stabilize GPR20 in the intermediate state, so that GPR20 could be quickly transformed into the fully active state in the presence of the G protein. Furthermore, an unidentified density, as will be discussed later, was observed in the transmembrane core of GPR20-Fab046 structure which might also impact the structure of the intracellular side of the GPR20 (Supplementary Fig. S8e).

In contrast to the intracellular side, the extracellular end of GPR20 does not exhibit notable changes between the GPR20-Fab046 and GPR20-Gi-Fab046 except for the flexible extracellular loops (Fig. 4c). Interestingly, ECL2 of GPR20 is very short and flexible which lacks a class-A conserved disulfide bond between ECL2 and TM3. This structural feature of ECL2 may be associated with the accommodation of the unique N-terminal cap which folds over next to the position of ECL2 in all three GPR20 structures (Figs. 2a and 4c).

### Binding interfaces for the antibody and G protein

**Fig. 5:**
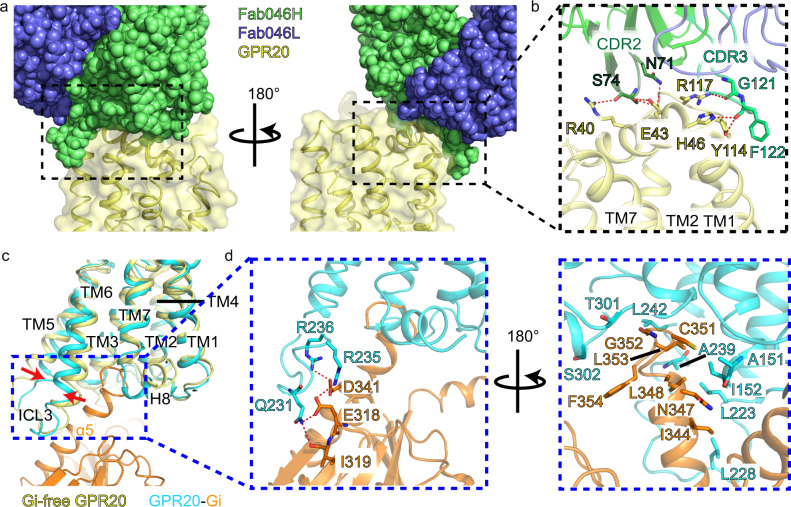
Binding interfaces for the Fab046 antibody and Gi protein on GPR20. **a** The binding interface between Fab046 (heavy chain in green, light chain in purple, spheres) and GPR20 (yellow, ribbon and transparent surface) is shown in two different views. **b** Magnified view of the antibody binding interface. CRD2 is shown in dark green, CDR3 is shown in light green, key residues are shown as sticks, and polar interactions are highlighted as red dashed lines. **c** The Gi binding interface. **d** Polar interactions (left) and hydrophobic interactions (right) between GPR20 (cyan) and Gi protein (orange). Key residues are shown as sticks, and polar interactions are highlighted as red dashed lines.

In both GPR20-Fab046 and GPR20-Gi-Fab046 structures, the antibody fragment stabilizes the complex through an antibody binding interface composed of CDR2 and CDR3 of Fab046 heavy chain and the extracellular side of GPR20 mainly consisting of the N-terminal cap, extracellular side of TM1 and ECL1 of GPR20 (Supplementary Fig. S9), which is consistent with the previous epitope mapping study that Ab046 mainly binds to the N-terminal domain and ECL1 of GPR20^17^. The interface is maintained by seven pairs of hydrogen bonds between Fab046 and GPR20: side chain of N71^CDR2^ with E43^N-term^, S74^CDR2^ forming two hydrogen bonds with E43^N-term^, backbone carbonyl of S74^CDR2^ with R40^N-term^, backbone carbonyl of G121^CDR3^ with R117^ECL1^, the main-chain carbonyl oxygen of F122^CDR3^ with Y114^ECL1^, backbone carbonyl of F122^CDR3^ with H46^N-term^; and one π-π interaction: F122^CDR3^ with Y114^ECL1^ (Fig. 5a-b; Supplementary Table S2). The molecular interactions identified between GPR20 and Fab046 may aid in the rational design/optimization of tool antibodies with different affinities or new functions for therapeutics development.

The structures of the GPR20-Gi complex with or without Fab046 show almost identical G protein coupling interface (Supplementary Fig. S7), which consists of TM2-3, TM5-6, ICL1-3 and H8 of GPR20, as well as the α5 helix of the Gα subunit: Q231^6.25^ forms hydrogen bonds with E318^h4s6.12^ and I319^S6.01^ in Gαi^34^, R235^6.29^ and R236^6.30^ form salt bridges with E318^h4s6.12^ and D341^H5.13^, and other hydrophobic interactions are formed by a series of hydrophobic residues (Fig. 5c, d; Supplementary Fig. S10a). Consistent with the structural findings, mutagenesis and cellular functional assays showed that most of the mutations at the interface reduced the signaling activity of GPR20 (Supplementary Fig. S10b). Compared to several representative GPCR-Gi structures including APJ^23^, CB_2_R^35^, D_2_R^36^, A_1_R^37^ and GAL_1_R^29^, where the αN helices of the Gαi subunits interact with the ICL2 of receptors to stabilize the GPCR-Gi protein complex, such a contact does not exist in GPR20 suggesting that it may utilize a slightly different mechanism for Gi coupling (Supplementary Fig. S10c, d).

## Discussion

Orphan GPCR research is still in its infancy; however new opportunities in this area are emerging given the increased number of receptor structures that have been reported and the diverse mechanisms for their constitutive activity uncovered. Here we report the structures of the orphan GPR20 with different states (GPR20-Gi, GPR20-Gi-Fab046 and GPR20-Fab046). These structures reveal a new mechanism to confer the orphan receptor’s high basal activity which might be attributed to the uniquely folded N-terminal α-helical cap region. Structural findings together with mutagenesis analysis suggest an “agonist-like” role of this N-terminal helix. In particular, the key residue F38^N-term^ is located right above a hydrophobic network in GPR20-Gi and GPR20-Gi-Fab046 complexes (Fig. 3c; Supplementary Fig. S7c, d), through which the activation signal can be transmitted to the toggle switch to trigger conformational changes at the intracellular side. We also report the structures of GPR20 in complex with the selective but non-functional antibody, which uncover the specific binding interface between GPR20 and Fab046, thus providing the accurate template for tool antibody discovery. Moreover, as GPR20 shows therapeutic potential in GIST and other intestinal disorders, our structures may offer opportunity for rational drug discovery targeting GPR20 for related diseases.

**Fig. 6:**
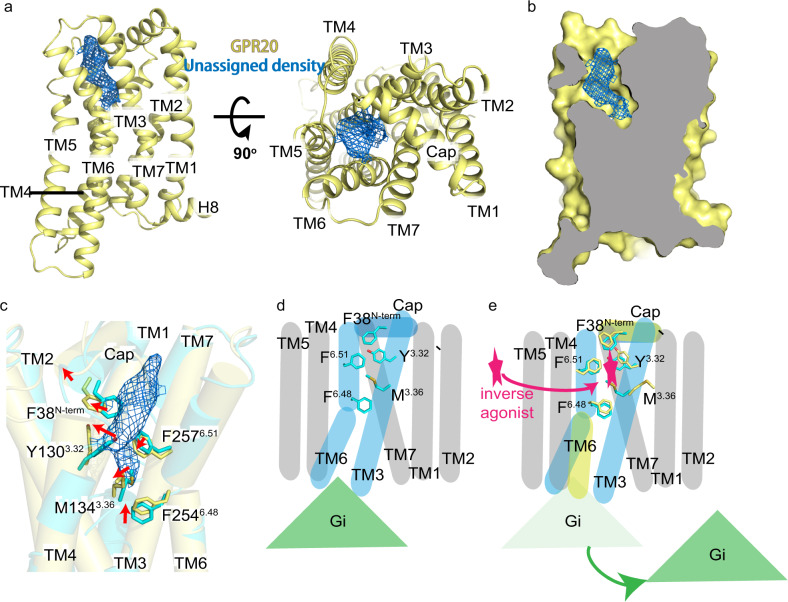
Unassigned density in the orthosteric pocket. **a** Two orthogonal views of the cartoon representation of GPR20 in yellow. The unassigned electron density observed in the canonical orthosteric pocket of GPR20 in the Gi-free GPR20-Fab046 complex is shown as a blue mesh. **b** A cross-section view of GPR20 in the GPR20-Fab046 structure (yellow) to show the shape complementarity of the unassigned electron density (blue mesh) with the orthosteric pocket. **c** Overlay between GPR20-Fab046 (yellow, unassigned density shown as a blue mesh) and GPR20-Gi-Fab046 (cyan) structures. Key residues of the hydrophobic network are shown as sticks. **d**, **e** Working model of putative activation mechanism for GPR20.

As mentioned above, an extra cryo-EM density in the core of the 7TM region was observed in the GPR20-Fab046 map (Fig. 6a, b). Structural superposition of this extra density of GPR20 with corresponding ligand positions from representative ligand-bound GPCRs—such as LPA in the LPA_1_R^27^, S1P in the S1P_1_R^27^, LSD in the 5-HT_2B_R^38^, BI-167107 in the β_2_AR^30^, and AM12033 in the CB_2_R^35^—shows that it overlays with the orthosteric ligand-binding pocket in these receptors (Supplementary Fig. S11a). Moreover, we identified several residues surrounding this density that may contribute to the formation of the pocket: F38^N-term^, Y130^3.32^, M134^3.36^, F254^6.48^, F257^6.51^, H258^6.52^, Y279^7.35^ (Supplementary Fig. S11b). Mutagenesis and cellular functional assays showed that mutating any single residue in this putative pocket reduced the basal activity of GPR20 (Supplementary Fig. S11c and Table S3), suggesting that these residues may constitute the orthosteric pocket and the density here may represent an unknown ligand. As it was previously reported that GPR20 might be closely related to lipid receptors^20^, we tried modeling the endogenous ligands from all known structures of lipid GPCRs into this unassigned density one by one, however, none of them could be fit into the density properly (data not shown). It is also worth mentioning that this density was only observed in the GPR20-Fab046 structure but not the other two Gi-coupled GPR20 complex structures reported in this study. Though we cannot rule out the caveat originated from the two stabilizing mutations (maintained around 50% of the basal activity relative to the WT protein, Supplementary Fig. S6a) in this specific construct, another possibility of assigning this density to an endogenously inverse agonist may merit future investigation.

To conclude, we can reconcile all the structural findings in this study in a following working model for GPR20 (Fig. 6c–e): in the absence of the endogenous ligand, GPR20 can couple with Gi proteins to form a constitutively active complex (referring to the two Gi-coupled GPR20 structures), which is achieved by the N-terminal cap especially the F38^N-term^ residue and the hydrophobic network that it engaged (Fig. 6d); when the ligand binds (referring to the unassigned density in the Gi-free GPR20 structure), the F38 is pushed upward away from the orthosteric pocket, followed by a disruption of the hydrophobic network where the key residue movements in the active-state structure would otherwise clash with the unassigned density (Fig. 6c, e). Thus the G protein is de-coupled and receptor activation is inhibited (Fig. 6e). Though this model requires identity of the unassigned density along with extensive experimental validations which are beyond the scope of current study, our structural findings together with mutagenesis data may provide a starting point for design of inverse agonist for therapeutic opportunity as well as to guide ligand discovery toward deorphanization for GPR20.

## Materials and methods

### Constructs and expression of GPR20 and Gi heterotrimer for cryo-EM study

The codon-optimized nucleotide sequence of human WT GPR20 (UniProt ID Q99678) was synthesized by GenScript. The human *GPR20* gene was subcloned into an expression vector pFastBac1 (Invitrogen) with the addition of a haemagglutinin signal peptide, Flag tag, and a thermostabilized *Escherichia coli* apocytochrome *b*562RIL (BRIL)^24^ at the N-terminus of the receptor gene as well as an HRV 3 C protease recognition site followed by a 10x His tag at the C-terminus. The human dominant-negative Gαi1 (DNGαi subunit was generated by introducing three mutations: S47N, G203A, A326S) and the human WT Gβ1γ2 subunits (codon-optimized and synthesized by GenScript) were cloned into a pFastBac1 and pFastBacDual (Invitrogen) vector, respectively. The Gi protein-bound GPR20 complexes were obtained by co-expressing the receptor, DNGαi and Gβ1γ2 in *Trichuplusia ni Hi5* insect cells (Invitrogen, B85502) using the Bac-to-Bac Baculovirus Expression System (Invitrogen). *Trichuplusia ni Hi5* cells were infected at a cell density of 2–2.5 × 10^6^ cells per mL with three separate virus (MOI = 5) preparations for GPR20, DNGαi and Gβ1γ2 at a ratio of 1:2:2. The infected cells were cultured at 27 °C for 48 h before collection by centrifugation, and the cell pellets were stored at −80 °C for future use.

### Expression and purification of scFv16

The codon-optimized nucleotide sequence of scFv16 was synthesized by GenScript and subcloned into an expression vector pFastBac1 with an 8x His tag at the C-terminus. The scFv16 used in this paper was the same as that used in the structures of the CB1-Gi-scFv16^35^. In brief, scFv16 was expressed in secreted form from *Trichuplusia ni* Hi5 insect cells and purified by Ni-NTA chromatography. The supernatant was incubated with Ni-NTA resin (GenScript) at 4 °C for 2 h. The resin was then loaded to a gravity column and washed with 15 column volumes (CV) of wash I buffer containing 20 mM HEPES (pH7.5), 100 mM NaCl and 10 mM imidazole; followed by 15 CV of wash II buffer containing 20 mM HEPES (pH7.5), 100 mM NaCl and 30 mM imidazole. The protein was eluted with elute buffer containing 20 mM HEPES (pH7.5), 100 mM NaCl and 250 mM imidazole. The elute was collected and further purified using a Superdex 200 10/300 column (GE Healthcare). Monomeric fractions were pooled, concentrated 10 mg/mL with a 10-kDa cut-off concentrator (Millipore), and flash frozen in liquid nitrogen, then stored at −80 °C for further use.

### Purification and formation of ligand-free GPR20-Gi complex

The cell pellets corresponding to 1 L GPR20-Gi co-expression culture were thawed and lysed in the hypotonic buffer of 10 mM HEPES (pH 7.5), 10 mM MgCl_2_, 20 mM KCl with EDTA-free complete protease inhibitor cocktail tablets (Roche). The GPR20-Gi complex was formed in membranes by addition of 1 unit of apyrase (NEB). The lysate was incubated overnight at 4 °C, and the supernatant was discarded after centrifugation at 40,000 rpm for 30 min. The complex was solubilized from membranes in the buffer containing 50 mM HEPES (pH 7.5), 100 mM NaCl, 1% (w/v) lauryl maltose neopentyl glycol (LMNG, Anatrace), 0.2% (w/v) cholesteryl hemisuccinate (CHS) (Sigma), 2 units of apyrase at 4 °C for 2 h. The supernatant was isolated by ultracentrifugation at 35,000 rpm for 30 min, and then incubated with TALON IMAC resin (Clontech) and 20 mM imidazole overnight at 4 °C. The resin was washed with 15 CV (column volumes) of washing buffer I containing 25 mM HEPES (pH 7.5), 100 mM NaCl, 5% (v/v) glycerol, 0.1% (w/v) LMNG, 0.02% (w/v) CHS, 30 mM imidazole, and 15 CV of washing buffer II containing 25 mM HEPES (pH 7.5), 100 mM NaCl, 5% (v/v) glycerol, 0.03% (w/v) LMNG, 0.006% (w/v) CHS and 50 mM imidazole. The protein was eluted using 3 column volumes of elution buffer containing 25 mM HEPES (pH 7.5), 100 mM NaCl, 10% (v/v) glycerol, 0.01% (w/v) LMNG, 0.002% (w/v) CHS and 250 mM imidazole. The eluate (GPR20-Gi complex) and scFv16 were mixed in a 1:1.5 ratio for 0.5 h, then concentrated and injected onto a Superdex200 10/300 GL column (GE Healthcare) equilibrated in the buffer containing 20 mM HEPES (pH 7.5), 100 mM NaCl, 0.00075% (w/v) LMNG, 0.00025% (w/v) glyco-diosgenin (GDN, Anatrace), 0.0001% (w/v) CHS, 100 μM TCEP. The complex peak fractions were collected and concentrated to 2.5 mg/mL with a 100-kDa cut-off concentrator (Millipore) for electron microscopy experiments.

### Constructs, expression, and purification of Fab046

We used the previously reported IgG046^17^ to generate a Fab fragment (Fab046, codon-optimized and synthesized by GenScript, containing intact light chain and part of heavy chain (VH + CH1) of IgG046). The light and heavy chains of Fab046 were cloned into a pFastBacDual vector. The *Trichuplusia ni Hi5* insect cells were infected with baculovirus at a density of 2 × 10^6^ cells per mL. Cells were grown at 27 °C and collected 48 h after infection. The cells were centrifuged at 2000 rpm for 30 min, and the 1 L supernatant was loaded onto a 2 mL Ni-NTA resin. The column was washed with 15 CV of wash buffer containing 20 mM Tris-HCI (pH 7.55), 150 mM NaCl, and 20 mM imidazole and the protein was eluted with the same buffer supplemented with 250 mM imidazole, the protein was collected and purified over gel filtration chromatography using a Superdex 200 10/300 column equilibrated in the buffer containing 20 mM Tris-HCI (pH 7.55), 100 mM NaCl, and 10% glycerol. Monomeric fractions were pooled, concentrated to 4 mg/mL with a 30-kDa cut-off concentrator (Millipore), and flash frozen in liquid nitrogen, then stored at −80 °C for further use.

### Constructs, expression, and purification of Fab046 bound Gi-free and Gi-coupled complex

For GPR20-Gi-Fab046, the complex protein was purified as described above for the GPR20-Gi complex, except that Fab046 was added during the purification process: the eluate (GPR20-Gi complex) and Fab046 were mixed in a 1:1.5 ratio for 6 h, then concentrated and injected onto a Superdex200 10/300 GL column (GE Healthcare) equilibrated in the buffer containing 20 mM HEPES (pH 7.5), 100 mM NaCl, 0.00075% (w/v) LMNG, 0.00025% (w/v) GDN, 0.0001% (w/v) CHS, 100 μM TCEP. The complex peak fractions were collected and concentrated to 2.5 mg/mL with a 100-kDa cut-off concentrator (Millipore) for electron microscopy experiments.

For GPR20-Fab046, to improve protein stability of GPR20 alone, two stabilizing mutations (L139^3.41^W and D293^7.49^N, which were essential for GPR20’s purification) were introduced based on the above construct for GPR20-Gi complex. We used the Bac-to-Bac baculovirus system in *Spodoptera frugiperda (Sf9)* cells for expression. These cells were infected with baculovirus (MOI = 5) at a density of 2 × 10^6^ cells per mL. Cells were grown at 27 °C and collected 48 h after infection. The cells were washed once with a low-salt buffer containing 10 mM HEPES (pH 7.5), 20 mM KCl, 10 mM MgCl_2_ and protease inhibitor cocktail (Roche), and three times with a high-salt buffer containing 10 mM HEPES (pH 7.5), 1 M NaCl, 20 mM KCl, 10 mM MgCl_2_ and protease inhibitor cocktail. Before solubilization, purified membranes were incubated with 2 mg/mL iodoacetamide (Sigma) at 4 °C for 0.5 h. The protein was extracted from the membrane by 50 mM HEPES (pH 7.5), 500 mM NaCl, 1.0% (w/v) LMNG and 0.2% (w/v) CHS and stirred for 2 h at 4 °C. After centrifugation, the supernatant was incubated with TALON IMAC resin (Clontech) at 4 °C overnight. Then the resin was washed with 15 CV of buffer I containing 50 mM HEPES (pH 7.5), 500 mM NaCl, 5% (v/v) glycerol, 0.05% (w/v) LMNG, 0.01% (w/v) CHS, 10 mM MgCl_2_ and 20 mM imidazole. Then the resin was washed with 10 CV of buffer II containing 25 mM HEPES (pH 7.5), 100 mM NaCl, 5% (v/v) glycerol, 0.01% (w/v) LMNG, 0.002% (w/v) CHS, 40 mM imidazole. Next, the protein was eluted with 3 CV of buffer III containing 25 mM HEPES (pH 7.5), 100 mM NaCl, 5% (v/v) glycerol, 0.005% (w/v) LMNG, 0.001% (w/v) CHS and 220 mM imidazole. The purified GPR20 protein was concentrated with a 50-kDa cut-off concentrator to around 2 mg/mL, mixed with Fab046 in a 1:1.5 mole ratio and incubated on ice overnight to form the complex. The mixture was loaded on a Superdex 200 10/300 column with a running buffer of 20 mM HEPES (pH 7.5), 100 mM NaCl, 0.00075% (w/v) LMNG, 0.00025% (w/v) GDN, 0.0001% (w/v) CHS, 100 μM TCEP. Peak fractions containing the GPR20-Fab046 complex were pooled and concentrated with a 100-kDa cut-off concentrator to 2 mg/mL for cryo-EM studies.

### Preparation of vitrified samples for Cryo-EM

In total, 3 μL of the purified samples (GPR20-Gi, GPR20-Fab046 and GPR20-Gi-Fab046) at a concentration of around 2 mg/mL were applied to glow-discharged 300-mesh Au grids (Quantifoil, R1.2/1.3). Excess sample was removed by blotting with filter paper for 3.5 s before plunge-freezing in liquid ethane using a FEI Vitrobot Mark IV at 100% humidity and 8 °C.

### Cryo-EM data collection

All datasets were collected on a Titan Krios 300 kV electron microscope (Thermo Fisher Scientifics, USA) equipped with a GIF Quantum energy filter (20 eV energy slit width, Gatan Inc., USA). All the GPR20 datasets were recorded by a K3 camera (Gatan) at a nominal magnification of 105,000 (calibrated pixel size: 0.832 Å/pixel) and 15 e^-^/pixel^2^/s. The movies were recorded using the super resolution counting mode by SerialEM which applied the beam image shift acquisition method with one image near the edge of each hole. A 50 µm C2 aperture was always inserted during the data collection period. The defocus ranged from −0.7 to −2.2 µm. For each movie stack, a total of 40 frames were recorded, yielding a total dose of 60 e^−^/Å^2^.

### Cryo-EM image processing

For GPR20-Gi complex, 4347 movies were recorded and processed with cryoSPARC v.3.3^39^. Patch motion correction was used for beam-induced motion correction. Then, contrast transfer function (CTF) parameters for each dose-weighted micrograph were estimated by patch CTF estimation. Only images with the highest resolution of less than 4 Å were selected for further processing. A total of 4129 images were selected for auto blob picking, and particles were extracted to do 2D classification. 2D class averages with diverse orientations and clear secondary features were selected as 2D templates for another round of autopicking process by cryoSPARC. A total of 386,819 particles were selected from good 2D classification to generate the initial models. These particles and initial models were used to do 3D classification in heterogeneous refinement in cryoSPARC. 256,813 particles were selected for the final homogeneous refinement followed by nonuniform refinement and local refinement in cryoSPARC, resulting in density map with nominal resolution of 3.14 Å for the GPR20-Gi complex (determined by gold-standard Fourier shell correlation (FSC), 0.143 criterion). Estimation of local resolution was performed in cryoSPARC.

For GPR20-Gi-Fab046 complex, 4443 movies were recorded and processed with cryoSPARC. Motion correction and CTF were applied and estimated as in the case of the GPR20-Gi complex. Only images with the highest resolution of less than 4 Å were selected for further processing. A total of 4247 images were selected for auto blob picking, and particles were extracted to do 2D classification. 2D class averages with diverse orientations and clear secondary features were selected as the 2D templates for another round of autopicking process by cryoSPARC. A total of 314,744 particles were selected from good 2D classification to generate the initial models. These particles and initial models were used to do 3D classification in heterogeneous refinement in cryoSPARC. 164,932 particles were selected for the final homogeneous refinement followed by nonuniform refinement and local refinement in cryoSPARC, resulting in density map with nominal resolution of 3.22 Å for the GPR20-Gi-Fab046 complex (FSC = 0.143). Estimation of local resolution was performed in cryoSPARC.

For GPR20-Fab046 complex, 4458 movies were recorded and processed with cryoSPARC. Motion correction and CTF were corrected and estimated as GPR20-Gi complex. Only images with the highest resolution of less than 4 Å were selected for further processing. A total of 4,145 images were selected to do auto blob picking and particles were extracted to do 2D classification. 2D class averages with diverse orientations and clear secondary features were selected as the 2D templates for another round of autopicking process by cryoSPARC. A total of 839,525 particles were selected from good 2D classification to generate the initial models. These particles and initial models were used to do 3D classification in heterogeneous refinement in cryoSPARC. 418,288 particles were selected for final homogeneous refinement followed by nonuniform refinement and local refinement in cryoSPARC, resulting in density map with nominal resolution of 3.08 Å for the GPR20-Fab046 complex (FSC = 0.143). Estimation of local resolution was performed with local resolution estimation in cryoSPARC.

### Cryo-EM model building and refinement

The homology models of GPR20 and Fab046 were initially generated by Alphafold^40^. For Gi trimer and scFv16, the model 6KPF (PDB)^35^ was chosen. Each part of the target models was docked into the electron microscopy density map using UCSF Chimera^41^. Then, these models were used for model building and refinement against the electron density map. Subsequently, the generated model was manually adjusted in Coot^42^ followed by automatic real space refinement in real space in Phenix^43^ for several iterations. The model statistics were validated using Phenix^43^. The final refinement statistics are provided in Supplementary Table S1.

### BRET2 assay

To measure the dissociation of Gαβγ heterotrimer directly, we applied the BRET2 assay system as reported before^21^. In brief, HEK293T cells were plated in a 6-well plate. After 2 h, cells were transiently co-transfected with plasmids encoding WT or mutated GRR20 together with Gi BRET probe (Gαi1-RLuc8, Gβ3, Gγ9-GFP2) using Lipofectamine 2000 reagent (Life Technologies). Adenosine A_2A_ receptor (A_2A_R) that does not couple to Gi proteins was used as a negative control, Apelin receptor (APJ) that couples to Gi proteins was used as a positive control for the Gi BRET assay. 24 h after transfection, cells were distributed into a 96-well microplate (30,000–50,000 cells per well) and incubated for additional 24 h at 37 °C. For the constitutive activity measurement, white backings (Perkin Elmer) were applied to the plate bottoms, the transfected cells were washed once with HBSS and supplemented with 100 µL of 5 µM coelenterazine 400a (Nanolight Technologies). Plates were read in EnVision plate reader (Perkin Elmer) with 410 nm (RLuc8) and 515 nm (GFP2) emission filters with an integration time of 1 s per well. The GFP2 emission to RLuc8 emission ratio was used to compute the BRET2 ratios. ΔBRET represent the change of bioluminescence resonance energy transfer value. ΔBRET = BRET ratio (GPCR with G protein sensor) - BRET ratio (only G protein sensor).

## Supplementary information

Supplementary Information

## Data Availability

Cryo-EM maps of the GPR20–Gi, GPR20-Gi-Fab046 and GPR20-Fab046 complexes have been deposited in the Electron Microscopy Data Bank under accession codes EMD-34984, EMD-34993 and EMD-34983, respectively. The atomic coordinates of the GPR20-Gi, GPR20-Gi-Fab046 and GPR20-Fab046 complexes have been deposited in the Protein Data Bank under accession codes 8HS3, 8HSC and 8HS2, respectively. All other data are available upon request to the corresponding authors.
