# Supplementary material for: The activation mechanism and antibody binding mode for orphan GPR20
Source: Cell Discov. 2023 Feb 28;9:23. doi: 10.1038/s41421-023-00520-8 (PMC9971246; doi:10.1038/s41421-023-00520-8)
Supplement: Supplementary file 1 — Supplementary Information [file 41421_2023_520_MOESM1_ESM.pdf]

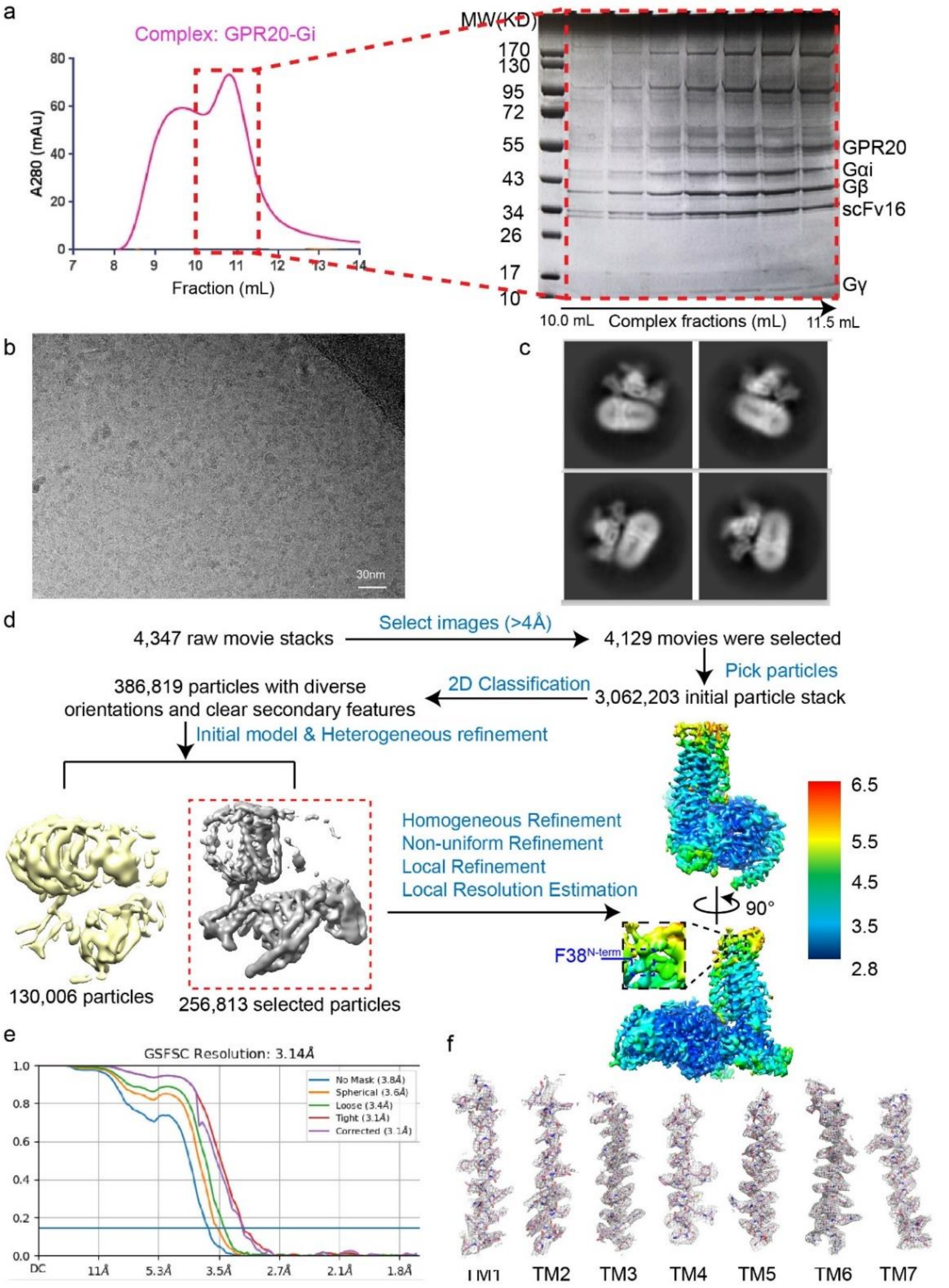

Supplementary Fig. S1 | Cryo-EM sample preparation and structure determination for GPR20–Gi complex.

a Left panel, analytical size-exclusion chromatography (SEC) of the purified GPR20-Gi protein complex, and, right panel, SDS–PAGE analysis of the complex sample after SEC. **b, c** Representative cryo-EM micrograph and reference-free two-dimensional class averages of the GPR20-Gi complex. **d** Workflow of cryo-EM data processing for GPR20-Gi complex. The final map is colored according to the local resolution. Density of key residue F38 is shown in blue box. **e** Gold-standard FSC curve, showing the overall nominal resolution at 3.14 Å. **f** EM maps for 7TMs of GPR20 in this structure.

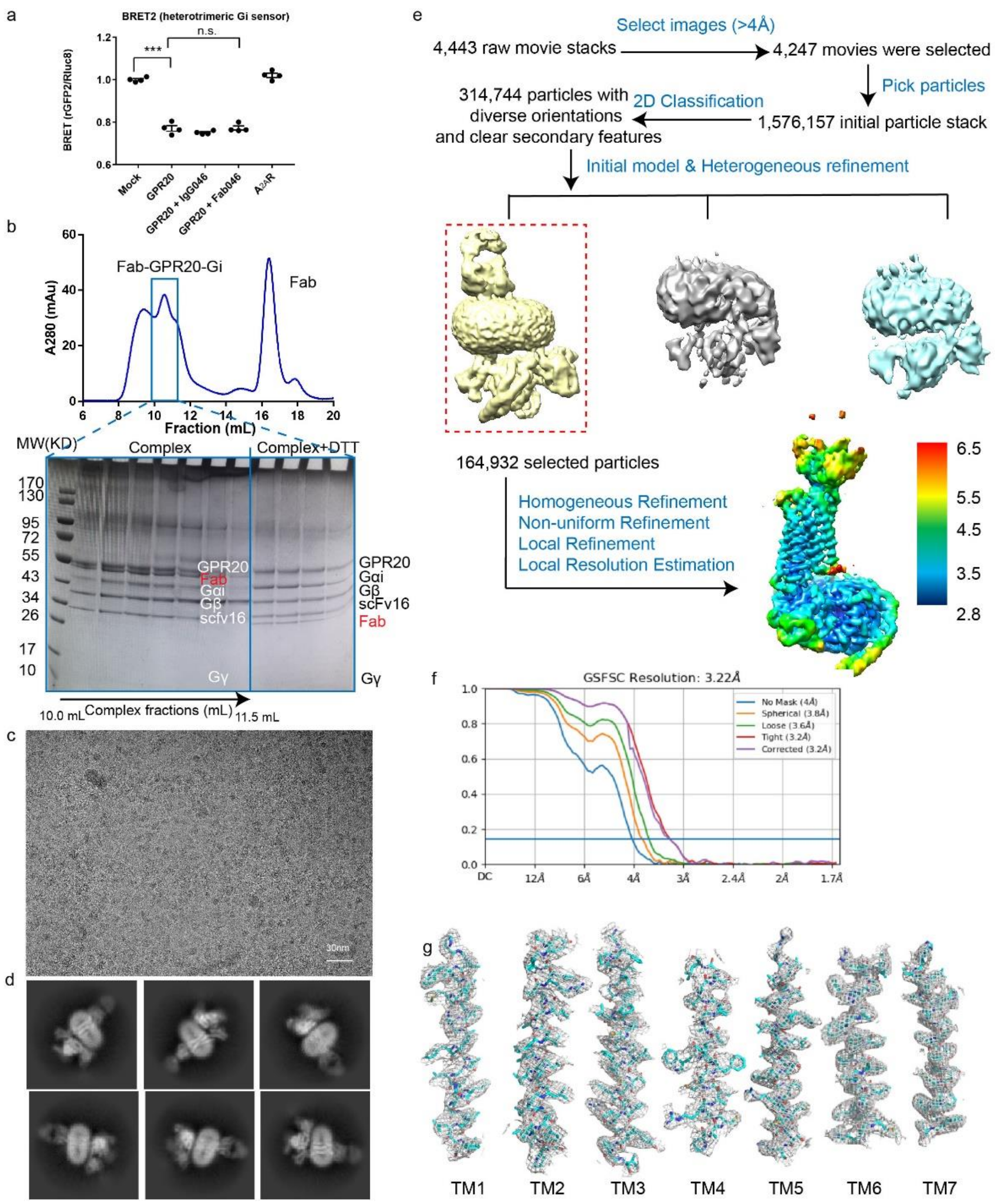

Supplementary Fig. S2 | Cryo-EM sample preparation and structure determination for GPR20-Gi-Fab046 complex.

a Normalized BRET values of HEK293 cells transiently co-transfected with the Gi BRET sensor along with either mock control, negative control ($A_{2A}R$) or GPR20. The activity of GPR20 in the absence or presence of Ab046 (in either IgG046 or Fab046 format) were measured. Data are normalized to the mock control and represented as the means ± s.e.m. for n=3 biologically independent experiments. Significance was determined by two-way analysis of variance (ANOVA) without repeated measures, followed by Dunnett's post hoc test (***P < 0.001; n.s., not significant). **b** Analytical size-exclusion chromatography (SEC) of the purified GPR20-Gi-Fab046 protein complex, and, SDS–PAGE analysis of the complex sample after SEC. **c, d** Representative cryo-EM micrograph and reference-free two-dimensional class averages of the GPR20-Gi-Fab046 complex. **e** Workflow of cryo-EM data processing for GPR20-Gi-Fab046 complex. The final structure is colored according to the local resolution. **f** Gold-standard FSC curve, showing the overall nominal resolution at 3.22 Å. **g** EM maps for 7TMs of GPR20 in this structure.

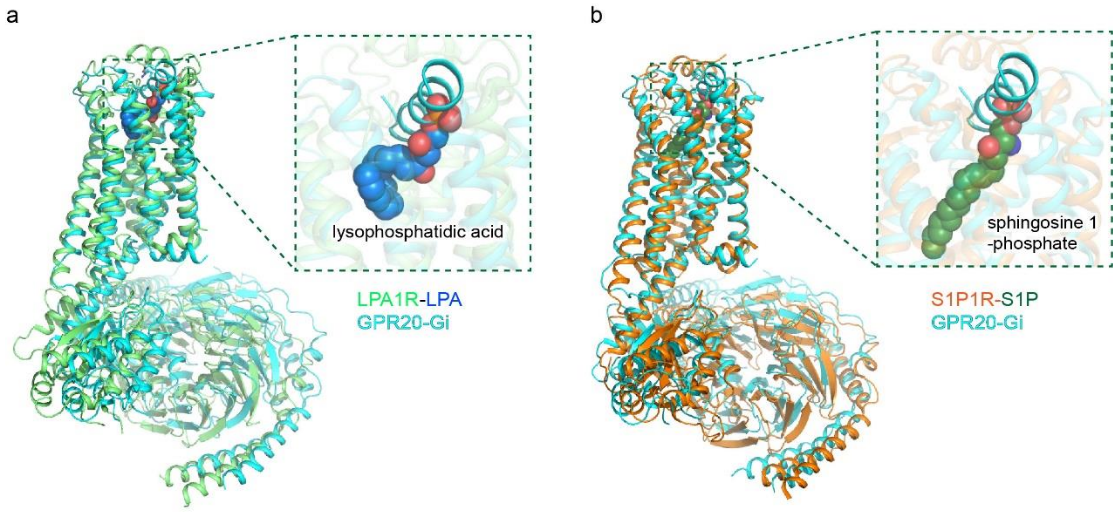

Supplementary Fig. S3 | Structural comparison of GPR20 with LPA_1R and $S1P_1R$.

a, b Structural comparison of GPR20-Gi (cyan) with LPA_1R-LPA-Gi (PDB: 7TD0, green-blue, **a**) and $S1P_1R$-S1P-Gi (PDB: 7TD3, orange-forest, **b**). N-terminal cap of GPR20 is shown as cyan cartoon. The ligands LPA and S1P are shown as spheres.

```
                       1       10        20        30        40
GPR20  ................MPSVSPAGPSAGAVPNATAVTTVRTNASGLEVPLFHLFARLDEE
GALR1  MELAVGNLSEGNASWPEPPAPEPGPLFGIGVEN...........................
GALR2  MNV.SGCPGAGN.......ASQAGGGGGWHPEA...........................

           50        60        70        80          90       100
GPR20  LHGTFPGLWLALMAVHGAIFLAGLVLNGLALYVFCCRT..RAKTPSVIYTINLVVTDLLV
GALR1  .........FVTLVVFGLIFALGVLGNSLVITVLARSKPGKPRSTTNLFILNLSIADLAY
GALR2  .........VIVPLLFALIFLVGTVGNTLVLAVLLR..GGQAVSTTNLFILNLGVADLCF

             110          120       130       140       150
GPR20  GLS.LPTRFAVY...YGARGCLRCAFPHVLGYFLNMHCSILFLTCICVDRYLAIVRPEGS
GALR1  LLFCIPFQATVYALPTWVLGAFICKFIHYF.FTVSMLVSIFTLAAMSVDRYVAIVHSRRS
GALR2  ILCCVPFQATIYTLDGWVFGSLLCKAVHFL.IFLTMHASSFTLAAVSLDRYLAIRYPLHS

      160       170       180       190
GPR20  RRCRQPACARAVCAFVWLAAGAVTLSVLGVTG........S...........RPCCRVFA
GALR1  SSLRVSRNALLGVGCIWALSIAMASPVAYHQGLFHPRASNQTFCWEQWPDPRHKKAYVVC
GALR2  RELRTPRNALAAIGLIWGLSLLFSGPYL..SYYRQSQLANLTVCHPAWSAPRR.RAMDIC

     200       210       220           230       240       250
GPR20  LTVLEFLLPLLVISVFTGRIMCALSRPG....LLHQGRQRRVRAMQLLLTVLIIFLVCFT
GALR1  TFVFGYLLPLLLICFCYAKVLNHLHKKLKNMSKKSEA..SKKKTAQTVLVVVVVFGISWL
GALR2  TFVFSYLLPVLVLGLTYARTLRYLWRAVDPVAAGSGARRAKRKVTRMILIVAALFCLCWM

         260       270        280       290       300       310
GPR20  PFHARQVAVALWPDMPHH.TSLVVYHVAVTLSSLNSCMDPIVYCFVTSGFQATVRGLFGQ
GALR1  PHHIIHLWAEF.GVFPLTPASFLFRITAHCLAYSNSSVNPIIYAFLSENFRKAYKQVFKC
GALR2  PHHALILCVWF.GQFPLTRATYALRILSHLVSYANSCVNPIVYALVSKHFRKGFRTICAG

          320       330       340              350
GPR20  HGEREPSSGDVVSMHRSSKGSGRHHILSAGPHALT.......QALA..............
GALR1  HIRKDSHLSDTKES.........KSRIDTPPSTNCTHV......................
GALR2  LLGRAPGRASGRVCAA.ARGTHSGSVLER.ESSDLLHMSEAAGALRPCPGASQPCILEPC

GPR20  .....NGPEA..........
GALR1  ....................
GALR2  PGPSWQGPKAGDSILTVDVA
```

Supplementary Fig. S4 | Sequence alignment of GPR20 with GAL1R and GAL2R.

Colors represent the similarity of residues: red background: identical; red text: strongly similar. The alignment was generated using Clustal Omega (https://www.ebi.ac.uk/Tools/msa/clustalo/) and the graphic was prepared on the ESPript 3.0 server (https://espript.ibcp.fr/ESPript/cgi-bin/ESPript.cgi).

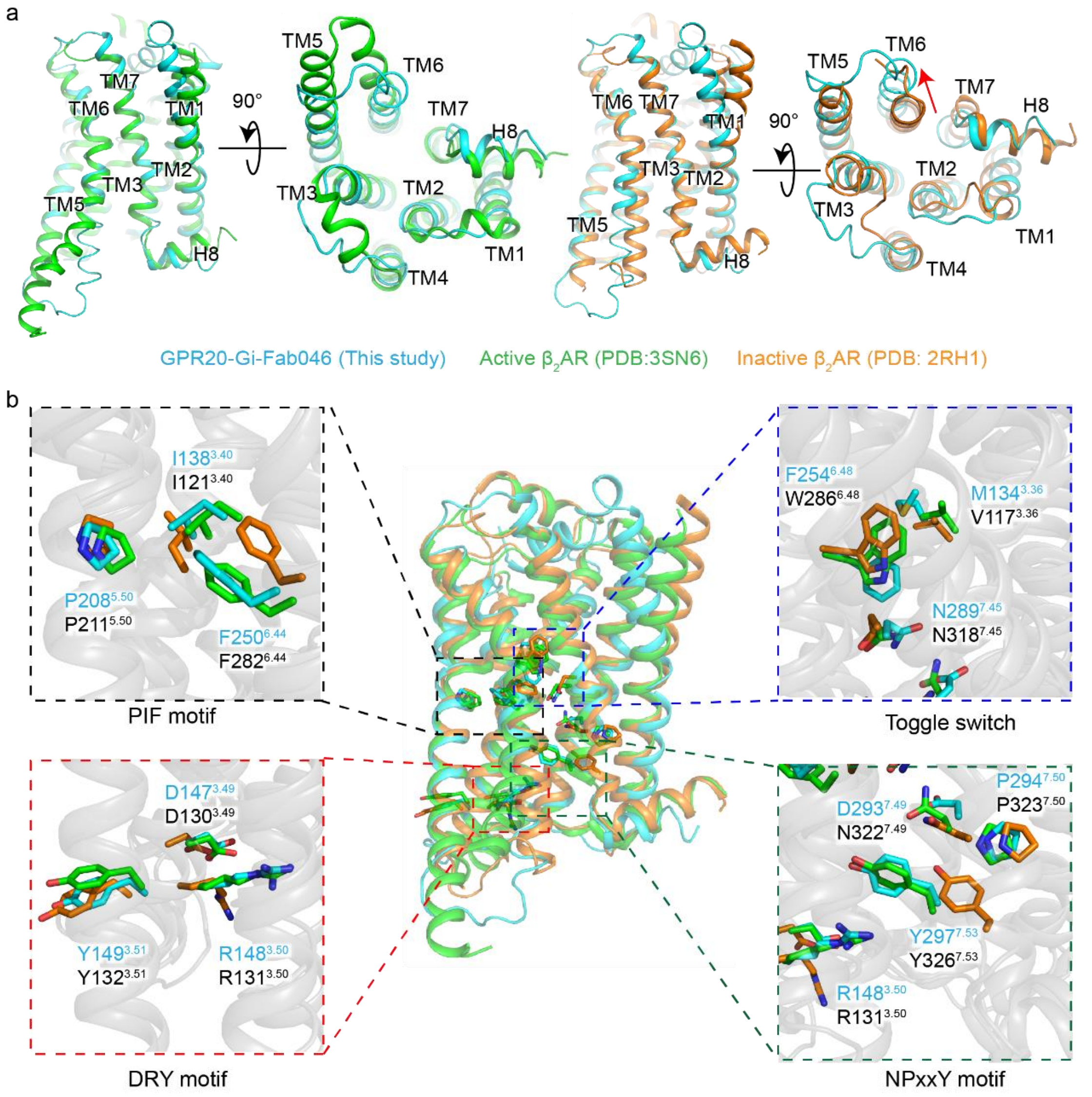

Supplementary Fig. S5 | Structural comparison of GPR20-Gi-Fab046 complex with active and inactive β2 adrenergic receptor (β$_2$AR).

a Side view and intracellular view of GPR20 with active (left, green) and inactive (right, orange) β$_2$AR. **b** The conformational rearrangement of residues in conserved "micro-switches". In contrast to β$_2$AR in the inactive state, the conformations of "micro-switch" such as the toggle switch, PIF, DRY, and NPxxY of GPR20 are shown as cyan sticks and resemble the conformations in the active β$_2$AR structure.

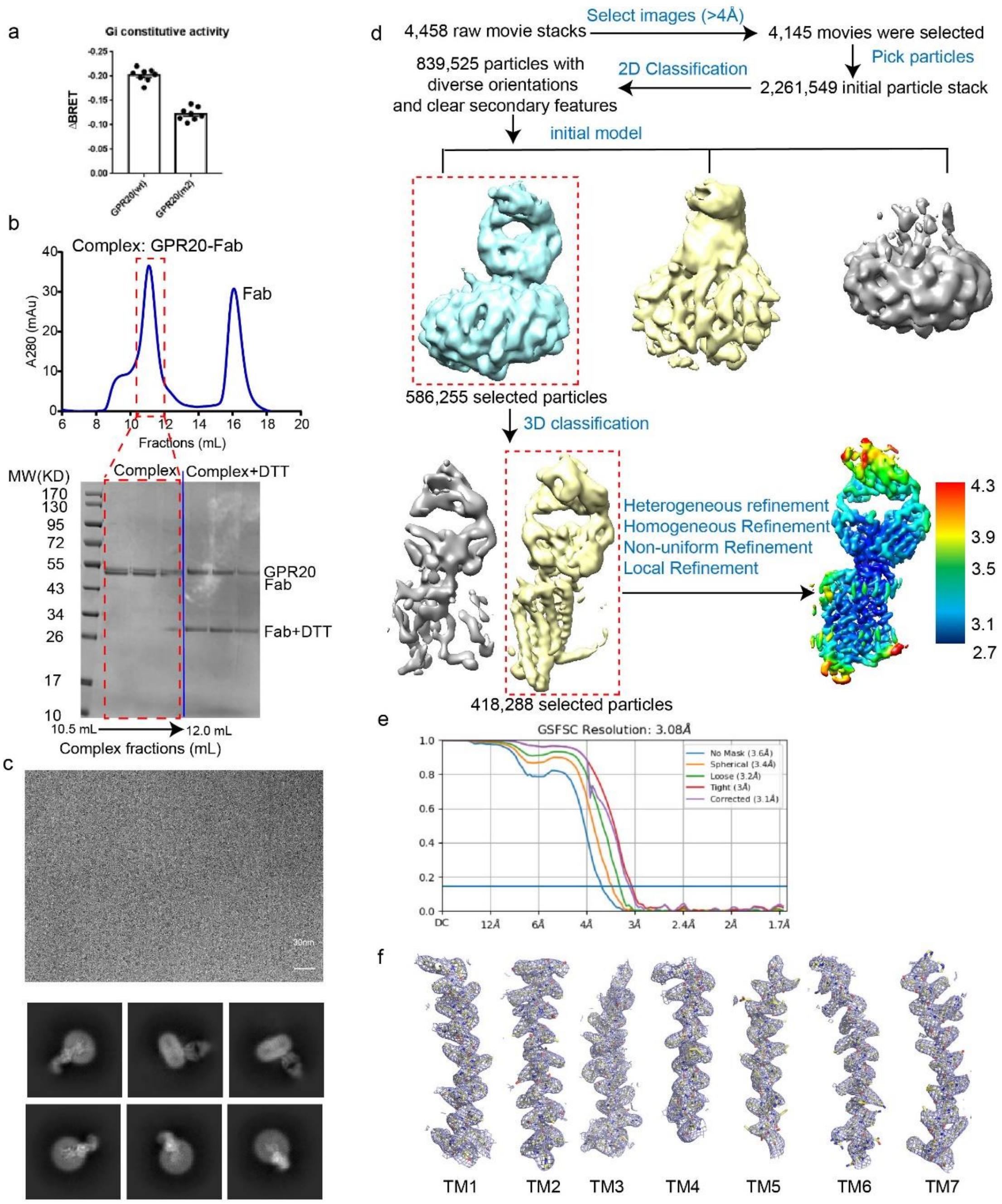

Supplementary Fig. S6 | Cryo-EM sample preparation and structure determination for Gi-free GPR20–Fab046 complex.

a The Gi constitutive activity of WT GPR20 and mutants are measured by BRET assay. GPR20(wt), wild-type GPR20; GPR20(m2), GPR20 contain L139$^{3.41}$W and D293$^{7.49}$N mutations. Data are mean ± s.e.m. (n≥3). **b** Analytical size-exclusion chromatography (SEC) of the purified GPR20-Fab046 protein complex, and, SDS–PAGE analysis of the complex sample after SEC. **c** Representative cryo-EM micrograph and reference-free two-dimensional class averages of the GPR20-Fab046 complex. **d** Workflow of cryo-EM data processing for GPR20-Fab046 complex. The final structure is colored according to the local resolution. **e** Gold-standard FSC curve, showing the overall nominal resolution at 3.08 Å. **f** EM maps for 7TMs of GPR20 in this structure.

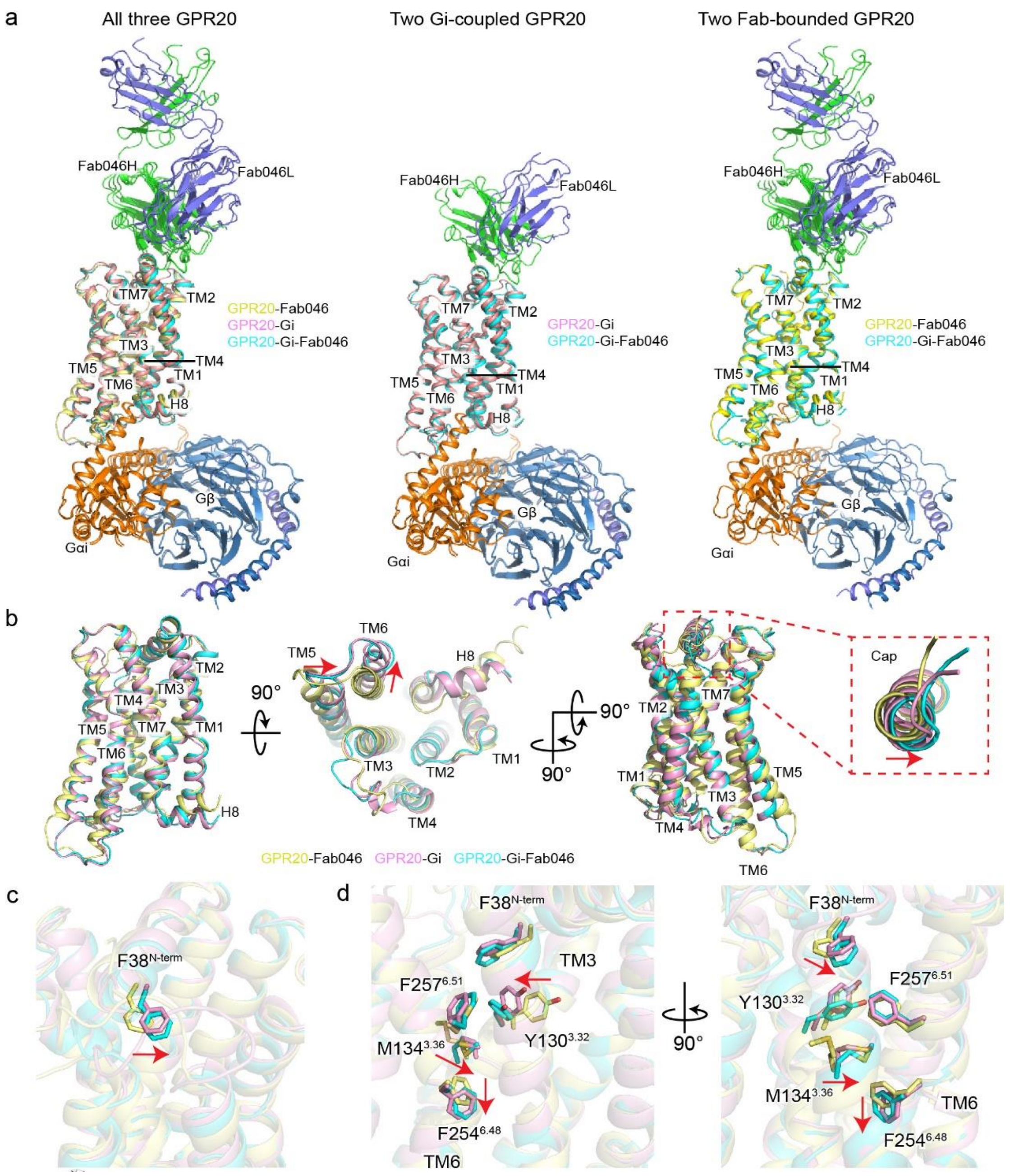

Supplementary Fig. S7 | Structural comparison between three GPR20 structures reported in this study.

a Overall comparison of three GPR20 structures by all components (left); comparison of two Gi-coupled GPR20 structures by all components (middle); comparison of two Fab046-bound GPR20 structures by all components (right). **b** Overlay of the three structures from different views. **c, d** Key residues involved in signal transmission from the N-terminal cap to the transmembrane region in three GPR20 structures.

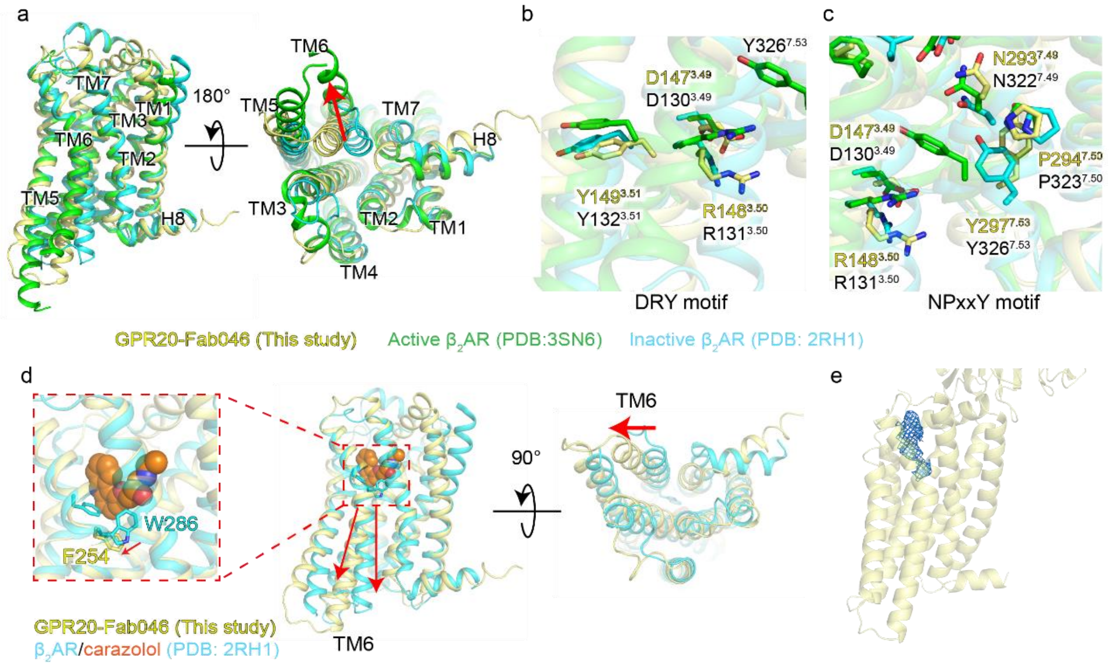

Supplementary Fig. S8 | Structural comparison of Gi-free GPR20-Fab046 with active and inactive β2 adrenergic receptor (β$_2$AR).

a Side view and intracellular view of GPR20 with active and inactive β$_2$AR. **b, c** The conformational rearrangement of residues in conserved "micro-switches". In contrast to β$_2$AR in the active state, the conformations of "micro-switch" such as the DRY (**b**) and NPxxY (**c**) of GPR20 are shown as yellow sticks and resemble the conformations in the inactive β$_2$AR structure (cyan). **d** Structural comparison of GPR20-Fab046 with inactive β$_2$AR. Key residues F254$^{6.48}$ of GPR20 and W286$^{6.48}$ of β$_2$AR were shown as sticks. Inverse agonist of β$_2$AR (carazolol) was shown as orange spheres. **e** The unidentified density observed in the transmembrane core of GPR20-Fab046 is shown as a blue mesh.

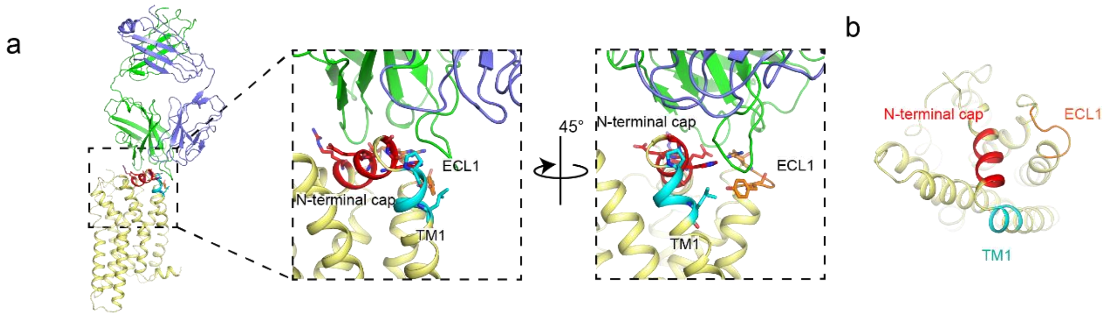

Supplementary Fig. S9 | Atomic model of GPR20-Fab046 to show the interface.

a, b The Fab046 binding interface is shown. The N-terminal cap that is involved in the interface is shown in red, the extracellular tip of TM1 in cyan and ECL1 in orange.

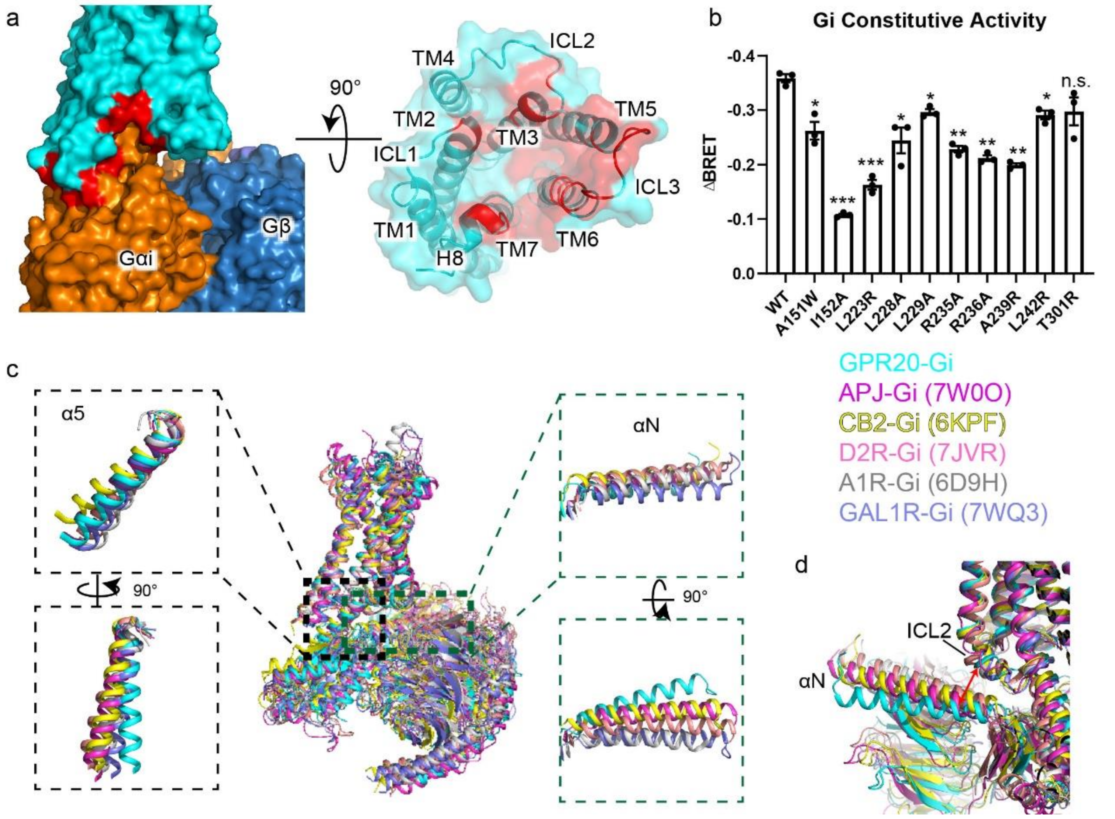

Supplementary Fig. S10 | Structural comparison of GPR20-Gi with several representative GPCR-Gi complexes.

a GPR20-Gi interface is shown in red. **b** The effects of Gi interface mutations on GPR20's constitutive activity (in **Fig. 5d**) are measured by BRET assay. Significance was determined by two-way analysis of variance (ANOVA) without repeated measures, followed by Dunnett's post hoc test (***$P < 0.001$, **$P<0.01$, *$P<0.05$, n.s.(not significant)). Data are mean ± s.e.m. (n = 3 independent biological experiments). **c, d** Superposition of the GPR20-Gi (cyan) with representative GPCR-Gi protein complexes: APJ-Gi (PDB 7W0O, purple), CB2-Gi (PDB 6KPF, yellow), D_2R-Gi (PDB 7JVR, pink), A1R-Gi (PDB 6D9H, grey) and GAL1R-Gi (PDB 7WQ3, slate). The α5 and αN helices in Gi proteins are highlighted in magnified views.

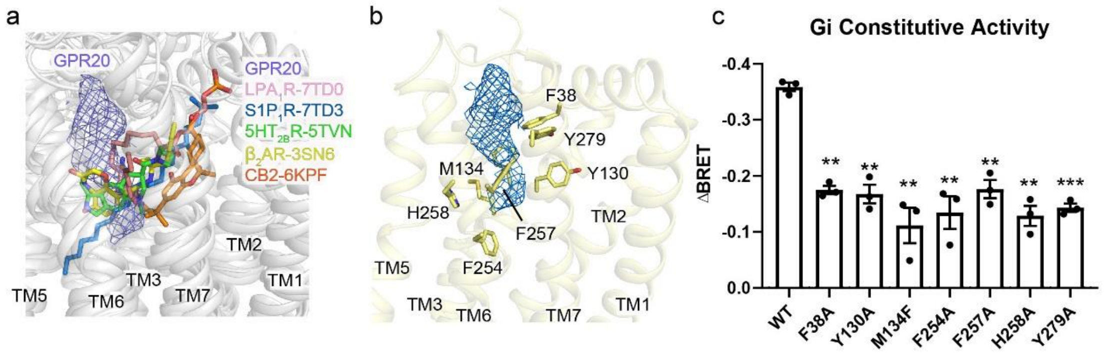

Supplementary Fig. S11 | Characterization of the unassigned density in Gi-free GPR20-Fab046 complex.

a Superposition of the unassigned electron density (blue mesh) with representative class-A GPCR ligands: LPA in LPA_1R (PDB 7TD0, pink), S1P in $S1P_1R$ (PDB 7TD3, blue), LSD in 5-$HT_{2B}R$ (PDB 5TVN, green), BI-167107 in β_2AR (PDB 3SN6, yellow) and AM12033 in CB2 (PDB 6KPF, orange). The six structures are superimposed and shown in grey cartoon. **b** Key residues of GPR20 surrounding the unassigned electron density are shown as sticks. **c** The effects of mutation of key residues (shown in **b**) are measured by BRET assay. Significance was determined by two-way analysis of variance (ANOVA) without repeated measures, followed by Dunnett's post hoc test (**P < 0.01, ***P < 0.001). Data are mean ± s.e.m. (n = 3 independent biological experiments).

Supplementary Table S1. Cryo-EM data collection, refinement and validation statistics

	GPR20-Gi	GPR20-Gi-Fab046	GPR20-Fab046
**Data collection and processing**			
Magnification	105,000	105,000	105,000
Voltage (kV)	300	300	300
Electron exposure (e-/ Å^2)	60	60	60
Defocus range (μm)	-0.7 to -2.2	-0.7 to -2.2	-0.7 to -2.2
Pixel Size (Å)	0.832	0.832	0.832
Symmetry imposed	C1	C1	C1
Initial particle images (no.)	3,062,203	1,576,157	2,261,549
Final particle images (no.)	256,813	164,932	418,288
Map resolution (Å)	3.14	3.22	3.08
FSC threshold	0.143	0.143	0.143
Map resolution range (Å)	2.8 ~ 6.5	2.8 ~ 6.5	2.7~ 4.3
**Refinement**			
Map sharpening B factor (Å^2)	-112	-110	-101
Model composition			
Non-hydrogen atoms	8,258	10,180	5,460
Protein residues	1,124	1,345	717
B factors (Å^2)			
protein	118.23	119.29	143.02
R.m.s. deviations			
Bond lengths (Å)	0.005	0.003	0.004
Bond angles (°)	0.751	0.707	0.713
Validation			
MolProbity score	1.77	1.73	1.53
Clash score	7.01	7.28	5.50
Poor rotamers (%)	0.00	0.00	0.00
Ramachandran plot			
Favored (%)	94.39	95.31	96.47
Allowed (%)	5.61	4.69	3.53
Disallowed (%)	0.00	0.00	0.00

Supplementary Table S2. Binding interface between GPR20 and Fab046

	Fab046	GPR20	pairs
Hydrogen bonds	$N71^{CDR2}$	$E43^{N\text{-}term}$	1
	$S74^{CDR2}$	$E43^{N\text{-}term}$	2
	$S74^{CDR2}$	$R40^{N\text{-}term}$	1
	$G121^{CDR3}$	$R117^{ECL1}$	1
	$F122^{CDR3}$	$Y114^{ECL1}$	1
	$F122^{CDR3}$	$H46^{N\text{-}term}$	1
π-π interaction	$F122^{CDR3}$	$Y114^{ECL1}$	1

The interface of GPR20-Fab046 is maintained by seven pairs of hydrogen bonds and one π-π interaction between Fab046 and GPR20: side chain of $N71^{CDR2}$ with $E43^{N\text{-}term}$, $S74^{CDR2}$ forming two hydrogen bonds with $E43^{N\text{-}term}$, backbone carbonyl of $S74^{CDR2}$ with $R40^{N\text{-}term}$, backbone carbonyl of $G121^{CDR3}$ with $R117^{ECL1}$, the main-chain carbonyl oxygen of $F122^{CDR3}$ with $Y114^{ECL1}$, backbone carbonyl of $F122^{CDR3}$ with $H46^{N\text{-}term}$; and one π-π interaction: $F122^{CDR3}$ with $Y114^{ECL1}$.

Supplementary Table S3. Basal signaling activity of wild-type GPR20 and mutants, measured by BRET assay

	Mutants	Gi activation of GPR20			Expression (% of WT)	
		ΔBRET	*P* value	n		*P* value
	WT	-0.3679±0.0095		3	100.0±4.3	
N-terminal cap	F38A	-0.1789±0.0057**	0.0010	3	99.5±1.8	0.8164
	L41E	-0.1913±0.0078**	0.0019	3	97.7±4.0	0.5389
	D42R	-0.1805±0.0092***	0.0006	3	98.8±5.5	0.8962
	L45E	-0.1720±0.0155**	0.0059	3	90.3±14.0	0.2987
	WT	-0.3590±0.0104		3	100.0±8.35	
Hydrophobic network	F38W	-0.3770±0.0276	0.4858	3	85.9±8.1**	0.0048
	F38A	-0.1749±0.0103**	0.0020	3	86.8±6.7*	0.0251
	Y130A	-0.1674±0.0237**	0.0033	3	90.5±7.4	0.2287
	M134F	-0.1114±0.0444**	0.0093	3	98.2±10.1	0.8513
	F254A	-0.1344±0.0411**	0.0092	3	105.9±4.7	0.4412
	F257A	-0.1763±0.0230**	0.0031	3	97.2±17.3	0.8002
	WT	-0.3590±0.0104		3	100.0±8.35	
Molecular switch	P208A	-0.1900±0.0123**	0.0089	3	91.6±17.0	0.4104
	F250A	-0.1638±0.0157**	0.0087	3	89.9±10.6	0.0713
	F254A	-0.1344±0.0411**	0.0092	3	106.0±4.7	0.4412
	Y297A	-0.1558±0.0175**	0.0077	3	92.1±23.3	0.6286
	WT	-0.3590±0.0104		3	100.0±8.35	
Gi interface	A151W	-0.2624±0.0228*	0.0288	3	86.8±21.7	0.3500
	I152A	-0.1045±0.0023***	0.0006	3	115.5±12.7	0.4029
	L223R	-0.1632±0.0127***	0.0004	3	103.2±15.2	0.7765
	L228A	-0.2449±0.0275*	0.0110	3	109.1±10.4	0.5471
	L229A	-0.2967±0.0079*	0.0129	3	110.8±8.7	0.1927
	R235A	-0.2286±0.0085**	0.0052	3	93.8±16.4	0.6080
	R236A	-0.2123±0.0074**	0.0031	3	110.7±6.9	0.2918
	A239R	-0.1991±0.0053**	0.0031	3	89.3±28.6	0.6377
	L242R	-0.2913±0.0111*	0.0416	3	108.1±24.0	0.5733
	T301R	-0.2980±0.0361	0.1914	3	100.4±3.7	0.9438
	WT	-0.3590±0.0104		3	100.0±8.35	
Orthosteric pocket	F38A	-0.1749±0.0104**	0.0020	3	86.8±6.7*	0.0251
	Y130A	-0.1674±0.0237**	0.0033	3	90.5±7.4	0.2287
	M134F	-0.1114±0.0444**	0.0093	3	98.2±10.1	0.8513
	F254A	-0.1344±0.0411**	0.0092	3	105.9±4.7	0.4412
	F257A	-0.1763±0.0230**	0.0031	3	97.2±17.3	0.8002
	H258A	-0.1287±0.0253**	0.0027	3	96.0±13.6	0.6318
	Y279A	-0.1431±0.0103***	0.0007	3	88.2±22.3	0.4744

Data are mean ± s.e.m. from at least three independent experiments (n). ***P < 0.001, **P < 0.01, *P < 0.05 by two-way analysis of variance (ANOVA) without repeated measures followed by Dunnett’s post hoc test compared to wild type. The comparable expression level of mutants (80%-120% of wild-type expression level) were achieved by adjusting the transfecting amounts of

plasmids encoding the respective receptor in HEK293T cells.
